# Supplementary material for: Comprehensive Annotation and Functional Exploration of MicroRNAs in Lettuce
Source: Front Plant Sci. 2021 Dec 24;12:781836. doi: 10.3389/fpls.2021.781836 (PMC8739914; doi:10.3389/fpls.2021.781836)
Supplement: Supplementary file 2 [file Data_Sheet_8.ZIP › GSM2230747.plot/Lsa-miRN1652a_Lsat_1_v5_gn_6_74641.1_227_TPlot.pdf]

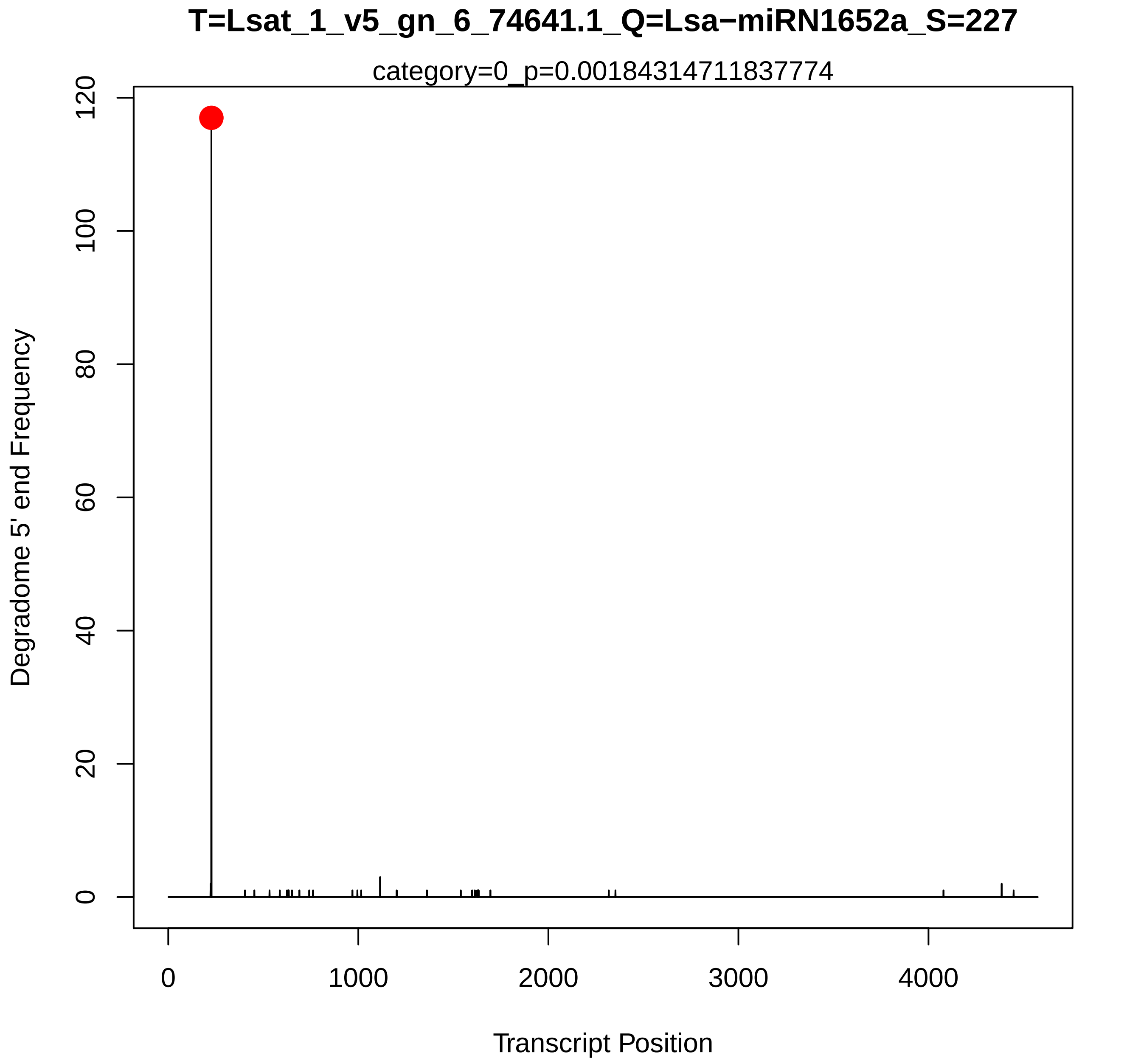
T=Lsat_1_v5_gn_6_74641.1_Q=Lsa−miRN1652a_S=227
category=0_p=0.00184314711837774
Degradome 5' end Frequency
0
20
40
60
80
100
120
0
1000
2000
3000
4000
Transcript Position
